# POLICY CONSIDERATIONS FOR GERONTOLOGY AND GERIATRICS CONTENT IN HEALTH PROFESSIONAL EDUCATION

**DOI:** 10.1093/geroni/igad104.0937

**Published:** 2023-12-21

**Authors:** Nancy Kusmaul

**Affiliations:** University of Maryland Baltimore County, Baltimore, Maryland, United States

## Abstract

Health professions educators in fields such as nursing, medicine, and social work struggle to prepare practitioners for the needs of growing numbers of older adults. In 2008, the National Academies of Science Engineering and Medicine identified that fewer than 5% of physicians choose to specialize in geriatrics, and even fewer in geriatric psychiatry, leading to significant shortages in mental health care for older adults. While other health professions such as clinical social workers or psychiatric nurse practitioners could potentially fill this gap, the story is the same in social work (4%) and nursing (1%). These numbers have improved marginally over the last fifteen years, but growing numbers of older adults have exacerbated shortages. In these professions, and in others, the model has been to have geriatrics/gerontology as a specialty, rather than integrated into the curriculum. Yet the interest in this specialization is not there and practitioners will have more exposure to this population than they might think. Members of the Social Research Practice & Policy section of GSA consider how we can shape policy to enhance geriatrics and gerontology education, and where AGHE can support health professions education (nursing, medicine, social work, etc.) and interdisciplinary health professional education in gerontology.

